# Glut1 Deficiency Syndrome (Glut1DS): State of the art in 2020 and recommendations of the international Glut1DS study group

**DOI:** 10.1002/epi4.12414

**Published:** 2020-08-13

**Authors:** Joerg Klepper, Cigdem Akman, Marisa Armeno, Stéphane Auvin, Mackenzie Cervenka, Helen J. Cross, Valentina De Giorgis, Adela Della Marina, Kristin Engelstad, Nicole Heussinger, Eric H. Kossoff, Wilhelmina G. Leen, Baerbel Leiendecker, Umrao R. Monani, Hirokazu Oguni, Elizabeth Neal, Juan M. Pascual, Toni S. Pearson, Roser Pons, Ingrid E. Scheffer, Pierangelo Veggiotti, Michél Willemsen, Sameer M. Zuberi, Darryl C. De Vivo

**Affiliations:** ^1^ Children's Hospital Aschaffenburg‐Alzenau Aschaffenburg Germany; ^2^ Department of Neurology and Pediatrics Vagelos College of Physicians and Surgeons at Columbia University New York NY USA; ^3^ Department of Nutrition Hospital Pediatria JP Garrahan Buenos Aires Argentina; ^4^ Department of Pediatric Neurology CHU Hôpital Robert Debre APHP Paris France; ^5^ Department of Neurology Comprehensive Epilepsy Center Johns Hopkins University School of Medicine Baltimore MD USA; ^6^ UCL NIHR BRC Great Ormond Street Institute of Child Health London UK; ^7^ Department of Child Neurology and Psychiatry IRCCS Mondino Foundation Pavia Italy; ^8^ Department of Neuropediatrics, Developmental Neurology and Social Pediatrics, Centre for Neuromuscular Disorders in Children, University Hospital Essen University of Duisburg‐Essen Essen Germany; ^9^ Department of Pediatric Neurology Paracelsus Medical Private University Nuremberg Germany; ^10^ Departments of Neurology and Pediatrics Johns Hopkins University Baltimore MD USA; ^11^ Department of Neurology Canisius Wilhemina Hospital Nijmegen The Netherlands; ^12^ Center for Motor Neuron Biology & Disease Departments of Neurology and Pathology & Cell Biology Columbia University Irving Medical Center New York NY USA; ^13^ Department of Pediatrics Tokyo Women's Medical University Tokyo Japan; ^14^ Matthew's Friends Charity & Clinics Lingfield UK; ^15^ Departments of Neurology and Neurotherapeutics, Physiology and Pediatrics Eugene McDermott Center for Human Growth and Development The University of Texas Southwestern Medical Center Dallas TX USA; ^16^ Mount Sinai Center for Headache & Pain Medicine New York NY USA; ^17^ First Department of Pediatrics Agia Sofia Hospital University of Athens Athens Greece; ^18^ Florey and Murdoch Institutes Austin Health and Royal Children's Hospital The University of Melbourne Melbourne Victoria Australia; ^19^ Pediatric Neurology V. Buzzi Hospital Child Neuropsychiatry University of Milan Milan Italy; ^20^ Department of Pediatric Neurology Radboud University Medical Centre Amalia Children's Hospital Nijmegen Netherlands; ^21^ Royal Hospital for Children & College of Medical Veterinary & Life Sciences University of Glasgow Glasgow UK

**Keywords:** children, consensus, diet, epilepsy, glucose transport, Glut1, Glut1 Deficiency Syndrome, Glut1D, Glut1DS, guideline, ketogenic

## Abstract

Glut1 deficiency syndrome (Glut1DS) is a brain energy failure syndrome caused by impaired glucose transport across brain tissue barriers. Glucose diffusion across tissue barriers is facilitated by a family of proteins including glucose transporter type 1 (Glut1). Patients are treated effectively with ketogenic diet therapies (KDT) that provide a supplemental fuel, namely ketone bodies, for brain energy metabolism. The increasing complexity of Glut1DS, since its original description in 1991, now demands an international consensus statement regarding diagnosis and treatment. International experts (n = 23) developed a consensus statement utilizing their collective professional experience, responses to a standardized questionnaire, and serial discussions of wide‐ranging issues related to Glut1DS. Key clinical features signaling the onset of Glut1DS are eye‐head movement abnormalities, seizures, neurodevelopmental impairment, deceleration of head growth, and movement disorders. Diagnosis is confirmed by the presence of these clinical signs, hypoglycorrhachia documented by lumbar puncture, and genetic analysis showing pathogenic *SLC2A1* variants. KDT represent standard choices with Glut1DS‐specific recommendations regarding duration, composition, and management. Ongoing research has identified future interventions to restore Glut1 protein content and function. **C**linical manifestations are influenced by patient age, genetic complexity, and novel therapeutic interventions. All clinical phenotypes will benefit from a better understanding of Glut1DS natural history throughout the life cycle and from improved guidelines facilitating early diagnosis and prompt treatment. Often, the presenting seizures are treated initially with antiseizure drugs before the cause of the epilepsy is ascertained and appropriate KDT are initiated. Initial drug treatment fails to treat the underlying metabolic disturbance during early brain development, contributing to the long‐term disease burden. Impaired development of the brain microvasculature is one such complication of delayed Glut1DS treatment in the postnatal period. This international consensus statement should facilitate prompt diagnosis and guide best standard of care for Glut1DS throughout the life cycle.

**Key Points**

- Early Glut1DS diagnosis is confirmed by characteristic clinical features, low CSF glucose, and pathogenic *SLC2A1* variants.
- Clinical features and treatment responses change with advancing patient age. Best outcomes correlate with early treatment.
- Age‐specific ketogenic diet therapies remain the standard of care.
- Continuing challenges are continuity of care during transition to adulthood, paroxysmal dyskinesias, intolerance to ketogenic diet therapies, and potential long‐term complications.
- Future therapies will focus on small molecule restoration of Glut1 protein/function, supplemental metabolic augmentation, and *SLC2A1* transfer strategies.

## 1 INTRODUCTION

Glucose is the essential metabolic fuel for brain. Glucose transport across the blood‐brain barrier (BBB) and astrocyte plasma membrane is exclusively facilitated by the glucose transporter type 1 (Glut1). Glut1 is primarily expressed in endothelial cells forming the BBB and in astrocytes, whereas Glut3 is primarily expressed in neurons. Consequently, a Glut1 genetic defect would impair glucose transport across the BBB and into astrocytes, resulting in a cerebral “energy crisis” termed glucose transporter type 1 deficiency syndrome (Glut1DS) (for comprehensive review see^1^). Patients classically present with infantile‐onset epilepsy, deceleration of head growth, impaired neurological growth and development, and complex movement disorders. Symptoms develop in an age‐specific pattern: Paroxysmal eye‐head movements and seizures are early presenting features in infancy. Developmental impairment becomes increasingly apparent and is followed by ataxia, paroxysmal exertion‐induced dystonia, and further movement abnormalities that develop over time often becoming the major symptoms in adolescents and adult Glut1DS patients. The principal diagnostic tool is a lumbar puncture showing low CSF glucose and low to low‐normal lactate concentrations in the setting of normal blood glucose and lactate concentrations. Most patients have autosomal dominant de novo heterozygous mutations in *SLC2A1* as the cause of the Glut1DS. Ketogenic diet therapies (KDT) provide a supplemental metabolic fuel for the brain and usually control seizures effectively. The beneficial effects on developmental delay and movement abnormalities, unfortunately, appear to be less striking.

Since the initial description of Glut1DS in 1991,^2^ the number of affected individuals has steadily grown facilitated by the advent of molecular diagnosis. A recently published prospective Scottish population‐based study reported a birth incidence of 1:24 000 presenting with epilepsy in the first three years.^3^ In line with a recent predicted incidence of 1.65‐2.22 per 100 000 births,^4^ this is likely a minimum incidence as cases may present later with epilepsy or movement disorders. In retrospective studies, the prevalence of Glut1DS was estimated to be 1:83 000 in Denmark^5^ and 1:90 000 in Australia,^6^ respectively. The estimated point prevalence of Glut1DS in Norway was 2.6/million inhabitants.^7^ All age groups from infants to adults are affected and present with age‐specific symptoms. Novel diagnostic tools and research strategies are emerging.

Recently, an international consensus on the optimal clinical management of children receiving dietary therapies for epilepsy was updated and serves as an excellent general guideline for KDT.^8^ Some issues relevant to Glut1DS were addressed in this publication. However, it has become clear that an international expert consensus focusing specifically on Glut1DS was required. This article highlights current Glut1DS knowledge and provides the first international consensus statement regarding the current standard of care for this condition.

## 2 METHODS

For the consensus guideline, the project lead authors, JK, EK, and DCD, identified other international experts for Glut1DS who have published on this entity either as first or senior authors in peer‐reviewed journals or have had extensive experience as dieticians (EN, BL) in the management of Glut1DS patients. A maximum of two authors per medical center was allowed resulting in 21 physicians and two dietitians from 10 nations (Europe: twelve, USA: eight, Argentina: one, Australia: one, and Japan: one). Each participant was asked to prepare a brief overview of the current state of the art for a specific Glut1DS topic. This expert opinion was then circulated to all authors for comments and revisions, surveys were circulated regarding controversial issues, and face‐to‐face consensus meetings were held during the 2nd European Glut1DS conference in East Grinstead, UK, on June 22, 2018, and the 8th biannual Glut1 Deficiency Foundation Conference on July 11, 2019, and July 12, 2019 in Washington DC, USA Authors were instructed to cite peer‐reviewed publications when available. In the absence of published literature, the participants were asked to base recommendations on their personal professional or collective medical center experience. Sections were collected, incorporated into a full document, and then emailed to the entire group for further review. For the surveys, 10 questions with 50 subheadings were emailed to 13 experts (one author per center) (see Data S1). Survey results were discussed at the 2nd European Glut1DS meeting. Additional international experts for specific Glut1DS‐associated topics were sought resulting in a total of 23 experts. Survey results were incorporated into the manuscript, providing percentage responses for individual topics. This process allowed for the sharing of expertise when the recommendation was controversial, and consensus was difficult to achieve. All participants reviewed and approved the final manuscript prior to submission.

## 3 STATE OF THE ART IN GLUT1DS AND CONSENSUS RECOMMENDATIONS

### 3.1 Clinical features in Glut1DS

Epilepsy: Pharmaco‐resistant seizures are frequently the first sign of Glut1DS. Any type of seizure can be observed.^9^ Generalized seizures are more frequent than focal seizures.^10^, ^11^ Early‐onset absence epilepsy (onset prior to age 4 years)^12^, ^13^ and epilepsy with myoclonic‐atonic seizures (Doose syndrome) have been associated with *SLC2A1* pathogenic variants. Any epilepsy associated with movement disorders should suggest Glut1DS. In contrast, catastrophic epilepsy syndromes such as Lennox‐Gastaut syndrome have not been reported. The initiation of KDT is highly effective in controlling seizures. Dietary treatment can achieve seizure freedom within a couple of days with normalization of EEG changes, frequently allowing for the withdrawal of any antiseizure medicines that might have been started before the correct diagnosis of Glut1DS had been established. Epilepsy tends to be the major clinical problem in infants and young children with Glut1DS, whereas seizures tend to decline or disappear in later childhood, adolescence, and adulthood.

Movement Disorders: In early infancy, distinctive paroxysmal eye‐head movements are the second most common initial sign of Glut1DS, after seizures.^14^, ^15^, ^16^ Episodes are typically involuntary and brief. The eye movements are repeated, multidirectional, saccadic, usually conjugate, and often accompanied by a head movement in the same direction. Later in childhood, other paroxysmal events emerge but the severity is highly variable. Presentation often involves some type of motor disturbance such as involuntary movements, ataxia, or weakness/paralysis. Awareness is generally preserved. Gradual clinical improvement, decreased frequency, and decreased severity of paroxysmal events are typical in adult life.^17^, ^18^, ^19^, ^20^, ^21^, ^22^, ^23^, ^24^, ^25^, ^26^, ^27^, ^28^, ^29^, ^30^ Paroxysmal nonmotor episodes include migraines, behavioral disturbances, cyclical vomiting, and sleep episodes.^19^, ^22^, ^24^, ^30^

In general, movement disorders are characteristic of Glut1DS. Severity ranges from minimal to severe. The movements can be persistent or paroxysmal, classically present preprandially, and are mitigated by meals. Persistent movement disorders include spasticity, ataxia, and dystonia often producing disturbances of gait, followed by chorea and tremor.^19^, ^24^, ^25^ Ataxia is increasingly evident in late infancy as the child stands and starts to walk.^25^ Ataxia is more truncal than appendicular. Chorea is often mild and involves the face and the distal upper limbs. A terminal intention tremor is frequent and often associated with other signs of cerebellar dysfunction. Myoclonus is generally epileptic; nonepileptic myoclonus is less common and includes startle myoclonus, action, and postural myoclonus.^17^, ^19^, ^27^ Dyspraxia is under‐recognized and includes oculomotor dyspraxia and oro‐buccal dyspraxia.^19^, ^28^ Paroxysmal movement disorders affect approximately 75% of patients and include paroxysmal eye‐head movements, paroxysmal exercise‐induced dyskinesia (PED), paroxysmal events manifested by major motor dysfunction, and paroxysmal events with complex neurological symptoms.^31^ These movement abnormalities are frequent and often precipitated by fasting and exercise.^18^, ^19^, ^20^, ^21^, ^26^, ^27^ Potential triggers are emotional stress, fever, fatigue, insufficient ketosis, sleep deprivation, temperature changes, and drugs.^17^, ^18^, ^19^, ^20^, ^21^, ^22^, ^26^, ^27^, ^28^, ^29^

Microcephaly may be acquired during infancy and is of varying degree likely correlating with clinical severity. ^32^

Development and cognitive function: Glut1DS is associated with mild‐to‐severe intellectual disability, with the degree of severity being proportional to the overall disease severity.^20^, ^26^, ^28^, ^33^ Dysarthria, with varying degrees of speech impairment, is observed in all affected individuals. Social adaptive behavior is an exceptional strength.^23^, ^25^ Performance skills usually are more affected than verbal skills, with prominent deficiencies in visuospatial and visuomotor abilities. Timing of KDT introduction is a predictive factor for cognitive outcome.^29^, ^34^ Early dietary treatment correlates with better intellectual and social adaptive skills.^25^

Atypical manifestations: Rare features described in Glut1DS include writer's cramp, intermittent ataxia, total body paralysis, Parkinsonism, and nocturnal painful muscle cramps in the legs.^32^ Alternating hemiplegia of childhood, hemiplegic migraine, cyclic vomiting, and stroke‐like episodes with paroxysmal hemiparesis, dysarthria, or aphasia have been described in individual patients.^23^, ^35^, ^36^ Other rare features include hemolytic anemia associated with PED, hepatosplenomegaly, periventricular calcifications, brain atrophy, pseudohyperkalemia, cataracts, and retinal dysfunction.^18^, ^37^

Adult Glut1DS: Data on adult Glut1DS are just emerging. Long‐term prognosis and long‐term adverse effects of KDT largely remain unknown. Changes in symptomatology over time include a shift from infantile‐childhood onset epilepsy to adolescent‐adult onset movement disorders including PED.^24^, ^25^, ^31^ Evaluation and treatment of adult Glut1DS also differ from pediatric Glut1DS. An extended metabolic evaluation may not be necessary, and for women of childbearing age, a pregnancy test should be considered prior to a KDT given that the risks of teratogenicity are unknown. Initiation of a KDT in adults is controversial given the side effects described in drug‐resistant childhood epilepsy including osteopenia, osteoporosis,^38^ potential cardiovascular risks,^39^, ^40^ and unclear effects on pregnancy. Also, energy and nutritional requirements in the mature brain are less than in the developing brain. In general, the modified Atkins diet (MAD) is considered a reasonable alternative for adolescents and adults^41^, ^42^ when treating poorly controlled seizures and paroxysmal movement disorders.

#### Committee conclusions

The Glut1DS phenotype becomes protean with advancing age. Clinical features consistent with Glut1DS include the following

- Any unexplained movement disorder with spasticity, dystonia, and ataxia,
- Complete seizure control by KDT in children with drug‐resistant epilepsy,
- Unexplained paroxysmal events at any age,
- Early‐onset absence epilepsy (under 4 years of age), and
- Myoclonic‐atonic epilepsy (Doose syndrome).

Experts rated the importance of clinical features for the diagnosis of Glut1DS as (a) an isolated clinical sign or (b) in combination with other neurological signs (survey question 2): Paroxysmal eye‐head movements in infancy were felt to be specific for Glut1DS (12 of 13 centers; 92%). In contrast, developmental delay otherwise unexplained, alternating hemiplegia, nonspecific paroxysmal events, and stroke‐like episodes were felt to be clinical features that rarely occurred as isolated clinical signs in this entity (Table 1).

**Table 1:** Ranking of clinical features specific for Glut1DS (question 2)

Clinical feature specific for Glut1DS that warrants lumbar puncture and *SLC2A1* analysis	Isolated	In combination
Paroxysmal eye‐head movements in infancy	12/13 (92%)	0/13 (0%)
Early‐onset absence epilepsy <4 y of age	10/13 (77%)	3/13 (23%)
Any complex movement disorder with spasticity, dystonia, ataxia as predominant features	6/13 (46%)	7/13 (54%)
Effective seizure control by KDT in children with drug‐resistant epilepsy	6/13 (46%)	7/13 (54%)
Myotonic‐atonic epilepsy (Doose syndrome)	6/13 (46%)	6/13 (46%)
Unexplained paroxysmal events (any age)	5/13 (38%)	8/13 (62%)
Early‐onset drug‐resistant childhood epilepsy unresponsive to antiseizure medication	4/13 (31%)	9/13 (69%)
Alternating hemiplegia	2/13 (15%)	10/13 (77%)
Stroke‐like episodes	2/13 (9%)	9/13 (69%)
Developmental delay otherwise unexplained	0/13 ( 0%)	12/13 (92%)

The potential to support the diagnosis of Glut1DS as either an isolated or combined clinical sign is shown as percentage of total (13 centers). Total numbers do not always add up to n = 13 as some questions were not answered by all centers.

### 3.2 Diagnosis of Glut1DS

EEG: In Glut1DS of all ages, an interictal EEG is often normal. Abnormalities appear more common at certain ages: In infants, focal slowing and epileptiform discharges are more prevalent, whereas in children ages two years or older 2.5‐ to 4‐Hz generalized spike‐wave pattern is observed.^43^ An intriguing feature, when present, is preprandial EEG abnormality that improves with feeding as glucose is restored to a starving brain.^44^

Lumbar puncture: Low CSF glucose values in the setting of normoglycemia, termed hypoglycorrhachia, represent the metabolic hallmark of Glut1DS.^45^ Other causes of hypoglycorrhachia such as hypoglycemia, meningitis, subarachnoid hemorrhage, or ventriculoperitoneal shunt systems need to be excluded.^46^, ^47^, ^48^ In Glut1DS, CSF lactate levels are always low‐normal or abnormally low, separating this condition from other diseases causing impaired brain energy metabolism, most notably mitochondrial diseases. The lumbar puncture should be performed in a postabsorptive state following a four‐ to six‐hour fast. Blood glucose measurements should be determined immediately prior to performing the lumbar puncture to avoid stress‐related hyperglycemia. Reference ranges are gender‐independent, but age‐specific.^49^ Hypoglycorrhachia in typical Glut1DS was originally defined as a cutoff value of 2.2 mmol/L (40 mg/dL).^2^, ^46^ In a retrospective analysis of 147 patients, CSF glucose levels ranged from 0.9 to 2.8 mmol/L (16.2 to 50.5 mg/dL) and CSF to blood glucose ratios ranged from 0.19 to 0.59.^45^ It is now clear that milder phenotypes may have CSF values of 2.2 to 2.9 mmol/L (41‐52 mg/dL), but never normal.^46^

Neuroimaging: Cranial magnetic resonance imaging (MRI) is useful to exclude structural and neurometabolic epileptic encephalopathies. One of four patients has abnormal nonspecific cranial MRI findings: hyperintensity of subcortical U‐fibers, prominence of perivascular Virchow spaces, and delayed myelination for age.^32^, ^50^, ^51^ 18F‐deoxyglucose‐positron emission tomography (^18^F‐FDG‐PET) can be a useful additional diagnostic tool. A diminished FDG signal emanating from the cerebellum, thalamus, and cerebral cortex, and an apparent increase in glucose accumulation in the striatum, particularly in the caudate nucleus, is the imaging signature of Glut1DS. The thalami display hypometabolism comparable to the degree of cortical depression. Within the cerebral cortex, a more pronounced uptake deficit is observed in the mesial temporal lobe.^29^, ^52^, ^53^ Magnetic resonance spectroscopy imaging has been applied in single cases of Glut1DS for diagnostic evaluation of brain energy metabolism but controlled studies are needed to assess diagnostic sensitivity and specificity.^54^, ^55^

Genetic analysis: Sequence analysis identifies heterozygous *de* pathogenic variants (or rarely, biallelic pathogenic variants) in *SLC2A1* in 81%‐89% of patients.^32^ Another 11%‐14% of patients are confirmed by deletion/duplication analysis.^32^ However, the absence of *SLC2A1* pathogenic variants does not always exclude Glut1DS. Pathogenic mechanisms may involve noncoding RNA genes as well as downstream defects in Glut1 translation, transcription, processing, activating, and trafficking (see Research section). *SLC2A1*‐negative patients can be diagnosed on the basis of hypoglycorrhachia and distinctive clinical features (Table 2), especially when responding favorably to KDT. Several specific de novo pathogenic *SLC2A1* variants affecting functional domains of the glut1 transporter have been identified. Also, pathogenic variants causing familial autosomal dominant and autosomal recessive inheritance have been reported in individual families.^56^, ^57^, ^58^ The type of genetic mutation often correlates with phenotypic severity: missense variants (mild and moderate severity); splice site and nonsense variants and insertions, deletions, and exon deletions (moderate and severe severity); and complete gene microdeletions (severe severity).

**Table 3:**
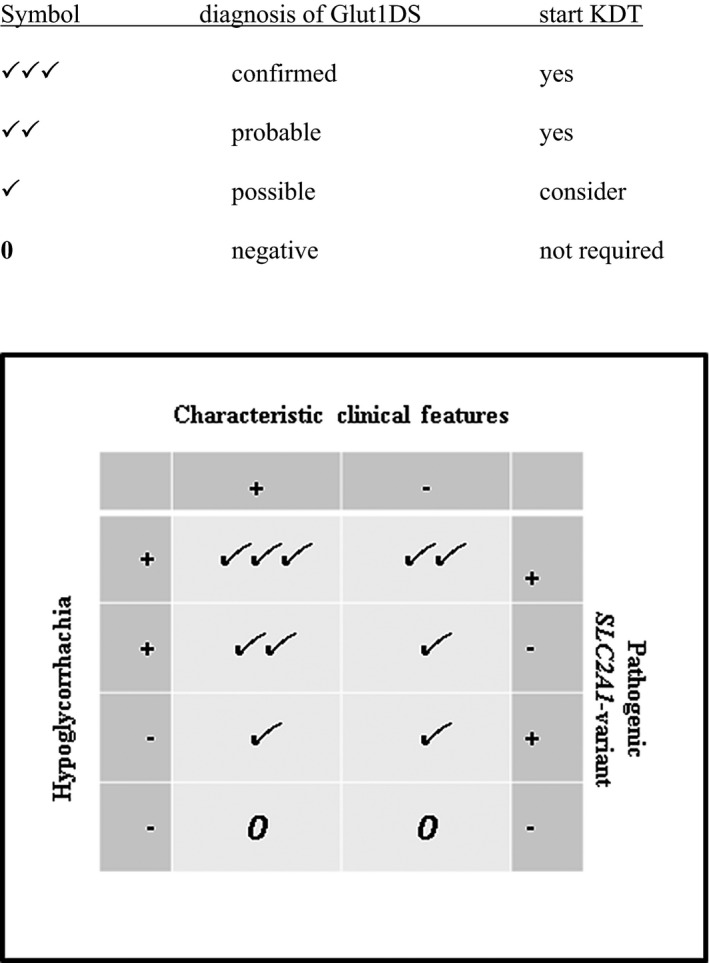
Consensus recommendation for Glut1DS diagnosis generated from survey results and discussions based on three key diagnostic criteria: characteristic clinical features, definite hypoglycorrhachia, and pathogenic <em>SLC2A1</em> variants

Laboratory studies: In the absence of pathogenic *SLC2A1* variants, an impaired 3‐O‐methyl‐D‐glucose uptake in erythrocytes between 35% and 74% of controls is diagnostic (for details see^32^). Western blot and oocyte expression analysis are additional tools to confirm pathogenicity. Recently, the measure of Glut1 present on the surface of the circulating red blood cells, as determined by flow cytometry (METAglut1^TM^ test), has been reported to be of diagnostic value for Glut1DS.^59^

#### Committee conclusions

Recommendations for Glut1DS diagnosis generated from the survey (13 centers, question 1) and expert discussions are shown in Table 2. All authors agreed that the definite diagnosis of Glut1DS requires the presence of characteristic clinical features, hypoglycorrhachia, and a pathogenic variant in *SLC2A1* (13/13, 100%). Controversies arose when some of these diagnostic criteria were negative or of unclear significance (survey question 1). In the presence of characteristic clinical features, most experts felt that two of three criteria were sufficient for the diagnosis. If the patient is asymptomatic, the clinical diagnosis is only justified, in the setting of hypoglycorrhachia and pathogenic *SLC2A1* variant, if the patient is a presymptomatic member in a familial Glut1DS setting.

Hypoglycorrhachia, epilepsy, movement disorders, and paroxysmal neurologic dysfunction may have other causes.^32^ Most centers perform brain MRI (10/13, 77%, and EEG/fasting EEG (8/13, 62%) to identify structural or epileptic encephalopathies. Opinions were mixed regarding metabolic parameters that should be measured (survey question 3): Blood lactate for mitochondrial disorders was determined in half the centers (7/13 54%), and basic blood parameters for renal, liver, and thyroid function and amino/organic acid analysis for metabolic disease were determined by just one‐third of centers (5/13, 38%). Brain PET (1/13, 8%) and fasting lipid analyses (0/13, 0%) were not considered standard investigations for the diagnosis of Glut1DS but were obtained in single centers. Glucose uptake studies by isolated erythrocytes also confirm the diagnosis but are not available on a commercial basis. The diagnostic value of the METAglut1^TM^ test is not yet established as a standard diagnostic tool for Glut1DS.

### 3.3 Treatment of Glut1DS

Ketogenic Diets: In Glut1DS, dietary therapy should be initiated as early as possible, providing the developing brain with a supplemental supply of metabolic fuel. Seizure control when managed with a classical KDT or MAD is good—one survey of Glut1DS families described 80% of patients with >90% seizure reduction including 64% of patients who no longer required antiseizure drugs.^60^ Ineffective seizure control in Glut1DS despite KDT has been reported.^29^, ^61^ Movement disorders and cognitive issues also improve with dietary KDT.^25^, ^29^, ^34^, ^47^ The general management of KDT for drug‐resistant childhood epilepsy and for Glut1DS is described in the revised international consensus.^8^ Specific aspects for KDT in Glut1DS are shown in Table 3. All patients with Glut1DS should monitor their ketosis by determination of beta‐hydroxybutyrate in capillary or venous blood. Urine ketones are a qualitative measure of acetoacetate and as such are not as adequate to quantitatively monitor ketonemia in Glut1DS patients.^32^, ^47^ Classic KDT typically provide higher levels of ketosis and may be preferred in younger children, especially those under age three years.^62^, ^63^ In adolescents and adults, the MAD may be more feasible for quality of life and compliance.^64^ The low glycemic index treatment (LGIT) provides very low ketones, has no evidence of benefit for Glut1DS, and is not recommended.^47^, ^65^ More effective KDT should be used in Glut1DS. If KDT are intolerable, increasing carbohydrates in regular diets has been described as an option.^66^, ^67^ Carnitine levels need to be monitored as patients may become secondarily carnitine‐deficient over time.^68^ Although most children with epilepsy on KDT can be transitioned off this treatment after two years, this plan is not appropriate for a child with Glut1DS. The benefits of KDT have no clear “end‐date” and are thought to extend into adulthood. ^69^

**Table 3:** Comparison of indication and treatment recommendations for KDT in drug‐resistant childhood epilepsy and Glut1DS

Criteria	KDT for drug‐resistant childhood epilepsy	KDT for Glut1 Deficiency Syndrome
Indication:		
Epilepsy	Insufficient seizure control by ≥two anticonvulsive medications	1st line treatment
Movement disorder	‐‐‐	1st line treatment
Development	‐‐‐	1st line treatment
Treatment		
Initiation	Optional	At diagnosis, any age, as early as possible
Duration	2 y +	Into adolescence/adulthood
Ketosis and KDT ratio	Variable	As high as tolerated
LGIT	Optional	Not recommended
Monitoring ketosis	Urine and blood ketones	Blood ketones
Carnitine levels	Optional	Recommended
Monitoring side effects	(+)	(+++)

Concurrent antiseizure drugs: Treating Glut1DS with antiseizure medications does not address the underlying metabolic defect. As a result, no individual drug can be recommended. In addition to being therapeutically ineffective, medications may do harm. Several examples were discussed. For example, carbonic anhydrase inhibitors such as acetazolamide, topiramate, and sulthiame may aggravate acidosis.^70^ Topiramate, acetazolamide, and zonisamide may increase the risk of urolithiasis, especially in combination with KDT.^71^ Decreased efficacy of KDT has been reported with the concomitant use of lamotrigine in childhood drug‐resistant epilepsy.^72^ Barbiturates, diazepam, sodium valproate, chloralhydrate, methylxanthine, and ethanol are nonspecific inhibitors of Glut1 function in vitro. No inhibitory effects in vitro were observed for carbamazepine and phenytoin.^73^, ^74^, ^75^, ^76^ Prolonged exposure to phenytoin and its metabolite 5‐(4‐hydroxyphenyl)‐5‐phenylhydantoin was shown to stimulate glucose transport in vitro by up to 30%‐60%.^76^ However, the implication of these in vitro data for the clinical setting remains unclear.

Ketone salts and ketoesters: Ketone salts and ketoesters may serve as a supplemental fuel for the brain without dietary restriction. Both compounds can be administered orally and can achieve ketone plasma levels equivalent to KDT.^77^ Sodium 3‐hydroxybutyrate has been used individually in metabolic diseases such as multiple acyl‐CoA‐dehydrogenase deficiency and specific glycogen storage diseases.^78^ Oral ketone salts are freely available, but daily volumes up to 400 grams taste unpleasant and may cause sodium overload. Triheptanoin provides 5‐carbon ketone body ketosis and an additional anaplerotic effect.^79^, ^80^ Despite promising initial reports,^55^, ^81^ a phase 3 study of triheptanoin (UX007) for the treatment of Glut1DS (ClinicalTrials.gov Identifier: NCT01993186) failed to meet its primary and secondary endpoints. A similar phase 3, randomized, double‐blind, placebo‐controlled, crossover study to assess the efficacy and safety of triheptanoin (UX007) in the treatment of movement disorders associated with Glut1DS (ClinicalTrials.gov Identifier: NCT02960217) was halted prematurely due to lack of efficacy (https://clinicaltrials.gov/ct2/show/ NCT02960217). Peer‐reviewed National Institutes of Health funded studies using standard clinical trial criteria also are currently underway (NCT03041363, NCT03181399, NCT03301532). Novel approaches are ongoing, targeting small molecules and other biologics to enhance Glut1 expression and function.^82^ Anecdotal reports have described individual benefits of acetazolamide and L‐DOPA as treatment for paroxysmal movement disorders in Glut1DS.^83^, ^84^, ^85^

#### Committee conclusions

Ketogenic diet therapies remain the treatment of choice for Glut1DS and should be started as early as possible (13/13, 100%, survey questions 4,5,8). In children under age two years, a classical 3:1 KDT is the treatment of choice. Most centers continue a classical KDT to obtain a high degree of ketosis to meet the energy demands of the developing brain. Most centers also believe that for adolescents, adults, and noncompliant patients, the MAD provides a good alternative to the classical KDT (12/13, 92%). LGIT is not recommended as treatment for Glut1DS (12/13, 92%). All centers recommend continuing the KDT for as long as tolerated by the patient. The updated recommendations of the international ketogenic diet study group for the optimal clinical management of children receiving dietary therapies for epilepsy^8^ provide an excellent guideline for initiation and management of KDT in Glut1DS (10/13, 77%). Issues specific to Glut1DS (survey question 8) are listed in Table 3. Key issues are the initiation of KDT as early as possible, essential blood ketone measurements targeting 2‐5 mmol/L for blood beta‐hydroxybutyrate, and the continuation of KDT into adolescence and adulthood. Supplements are essential in KDT, but recommendations for routine carnitine supplementation were controversial: Most centers check carnitine levels at regular intervals, while others supplement carnitine routinely (survey question 9,10).

Current data on antiseizure medication in Glut1DS are controversial and insufficient. The committee was concerned about the possibility of doing harm with the sole use of antiseizure medications. Survey results showed that add‐on antiseizure drugs in combination with KDT used in centers are levetiracetam (9/13, 69%) and valproic acid (9/13, 69%; not in girls beyond menarche), followed by lamotrigine (5/13, 38%, survey question 6). Some centers considered ethosuximide, carbamazepine, oxcarbazepine, and zonisamide to be unhelpful. Currently, there is no basis to recommend any antiseizure drug in the management of Glut1DS, and there are concerns regarding potential harmful interactions with KDT Paroxysmal events unresponsive to antiseizure medication have been treated with alpha‐lipoic acid (2/13), triheptanoin (8/13), and acetazolamide (12/13) on an intention‐to‐treat basis (survey question 7). No recommendations can currently be made regarding effective PED treatment or the use of oral ketones or ketoesters.

### 3.4 Glut1DS management and research

Follow‐up. All patients with Glut1DS should be seen at regular intervals to monitor KDT, to address individual issues, and to share novel developments in the field.^8^, ^32^, ^47^ Follow‐up needs to be age‐specific as symptoms change from primarily seizures in infancy‐early childhood to movement disorders such as dystonia and paroxysmal exertional dyskinesias in adolescence‐adulthood.^25^ Cognitive dysfunction persists throughout life but there is no evidence for progressive worsening.^34^ Long‐term adverse effects of KDT such as growth impairment, nephrolithiasis, and cardiovascular risks need to be monitored at regular intervals.^8^

With patients approaching adulthood, it is critical to develop a transitional plan for diet therapy and for medical care to transition from a pediatric to an adult subspecialist.^41^

Research. A family of hexose and MCT transporters provides metabolic fuel for the brain. At the cellular level, Glut1 molecules function as tetramers^86^ to facilitate glucose transport across tissue barriers. The impact of Glut1DS on these systems is complex and the subject of ongoing research. *SLC2A1* variants could destabilize GLUT1 native interactions, generate novel interactions, trigger protein misfolding, and enhance protein aggregation.^87^ Glut1DS could be influenced by noncoding RNA genes^88^, ^89^ as well as downstream defects in Glut1 translation, transcription, processing, activating, and trafficking.^90^, ^91^ Research into disease mechanisms has identified novel targets for therapy by focusing (a) on the link between the fundamental metabolic defect (ie, brain glucose depletion or neuroglycopenia) and its metabolic and neurophysiological consequences, (b) on the mechanisms of transporter dysfunction,^79^, ^92^, ^93^ and (c) on the MRI and PET‐based investigation of human brain metabolism.^94^ These ongoing studies are enabling the development of supplemental metabolites to compensate for the neuroglycopenia as therapeutic strategies.^80^ Glut1 haploinsufficiency arrests brain angiogenesis during brain development, and the diminished brain capillary network further aggravates neuroglycopenia.^95^ Approaches to restore Glut1 protein content and function include upregulation of the normal *SLC2A1* allele using small molecule strategies or gene transfer strategies whereby a normal *SLC2A1* gene is delivered in a suitable viral vector to the Glut1‐deficient patient.^82^ Preclinical experiments in Glut1DS model mice using AAV9 vectors have demonstrated that gene replacement is effective and durable: Brain Glut1 expression and CSF glucose concentrations increase, brain growth and volume are maintained during development, motor performance is preserved, seizure activity is controlled, and brain angiogenesis proceeds normally.^82^, ^95^, ^96^ In contrast to successful presymptomatic or early symptomatic treatment, gene replacement in adult mice failed to improve symptoms, suggesting a therapeutic window of opportunity. This observation in Glut1DS and other neurodevelopmental disorders emphasizes the fundamental importance of newborn screening for such genetically determined conditions to facilitate early postnatal diagnosis and proactive treatment before the onset of symptoms and irreversible damage to the developing brain.

#### Committee conclusions

There was general agreement that patients should be monitored regularly for long‐term side effects of KDT such as kidney stones, growth retardation, and cardiovascular disease (blood pressure, fasting lipid profiles, transcranial Doppler ultrasound from age 10 years). Long‐term use of KDT in Glut1DS might generate adverse effects that might be more evident than shorter term use of KDT in drug‐resistant childhood epilepsy. Discontinuing KDT, the current standard of care for Glut1DS, in favor of clinical trials that are designed to investigate novel treatment considerations was rejected strongly by the group (12/13, 92%).

## 4 DISCUSSION

The increasing complexity of Glut1DS, since its original description in 1991,^2^ has highlighted the need to develop an expert consensus based on questionnaires, international conferences, and round table discussions. Experts agreed that clinical signs suggestive of Glut1DS warrant a prompt diagnostic workup. A properly controlled diagnostic lumbar puncture plus *SLC2A1* analysis remain critical to confirm Glut1DS. Normoglycemia, hypoglycorrhachia, and a low to normal CSF lactate concentration are necessary biomarkers for the diagnosis. KDT remain the standard of care and represent the best treatment for Glut1DS paroxysmal manifestations. The choice of KDT is influenced by several factors including patient age and tolerability.^8^ The importance of early diagnosis and treatment cannot be exaggerated. In classical cases of Glut1DS, the KDT should be started as early as possible postnatally with the highest degree of ketosis maintained to mitigate brain energy deficiency by providing optimal concentrations of metabolic fuels (glucose and ketone bodies) to the developing brain. It is less clear whether this biological rule applies to the milder phenotypes but experience with other genetic diseases suggests that it does. Deceleration of head growth during early infancy is an ominous sign of irreversible brain dysfunction.

Controversies arose, as expected, because of the lack of data on unresolved issues regarding Glut1DS. Centers have had to develop individual protocols that remain to be modified on the base of this consensus and future research studies. Disagreements revolved around basic issues such as the normal range of CSF glucose concentrations, *SLC2A1* variants of unclear significance, asymptomatic family members diagnosed with *SLC2A1* variants, and the use of KDT in pregnant Glut1DS patients. Treatment decisions are less clear in atypical variants of Glut1DS, especially in oligosymptomatic or late‐onset patients, because of limited experience. Newborn screening for this treatable disease is essential but remains to be established.

Despite the effectiveness of KDT, there is an unmet need in Glut1DS for additional therapies and novel approaches. Unresolved treatment issues involved the concomitant use of antiseizure drugs, compounds such as acetazolamide, cannabidiol, ketone salts, and ketone esters, and the pharmacological treatment of paroxysmal events, dystonia, and dysarthria that significantly impair patients' quality of life. Transition to adult medicine is often difficult for patients and treating physicians. It remains unclear how long dietary treatment should be continued in the management of adult Glut1DS and how long‐term adverse effects should be monitored and addressed.^32^, ^69^ Future research also needs to address the impact of Glut1 deficiency on other organs rich in Glut1 transporters such as heart, muscle, placenta, and retina.^97^, ^98^, ^99^, ^100^ The finding of impaired cerebral angiogenesis during development in Glut1DS model mice emphasizes the importance of early diagnosis and proactive treatment to prevent irreversible brain damage. Future therapies for Glut1DS are focusing on supplemental brain metabolic fuels, *SLC2A1* transfer, and small molecules designed to enhance Glut1 expression or activity.^82^

This first consensus emphasized the phenotypic heterogeneity of Glut1DS and key issues surrounding diagnosis, treatment, and long‐term management. Discussions highlighted areas of consensus, topics of controversy, and future challenges. These management guidelines are primarily meant to be informative as it relates to the current state of the art. The consensus committee fully expects that these guidelines can be superseded when dictated by individual unique patient circumstances. The committee also recognized the ongoing demand for optimal clinical care of the aging Glut1DS patients and the continuing need to improve transitional care to optimize long‐term management and treatment throughout the life cycle.

## Supporting information

Data S1
