# Supplementary material for: Glut1 Deficiency Syndrome (Glut1DS): State of the art in 2020 and recommendations of the international Glut1DS study group
Source: Epilepsia Open. 2020 Aug 13;5(3):354–65. doi: 10.1002/epi4.12414 (PMC7469861; doi:10.1002/epi4.12414)
Supplement: Supplementary file 1 — Data S1 [file EPI4-5-354-s001.pdf]

Glut1D consensus: Questions for Discussion (2018):

Please highlight/bold/or underline your answers and re-email back to me (joerg.klepper@klinikum-ab-alz.de). Please remember – only one survey per center. Thanks!

1. Glut1D is diagnosed on a) clinical features, b) LP: hypoglycorrhachia with normal to low CSF lactate, and c) genetics: mutations in the SLC2A1 gene.

Please tick × for the combination of diagnostic criteria that are the minimum necessary to **confirm** Glut1D:

Clinical features	LP	genetics	confirms diagnosis of Glut1D (multiple × possible)
a			
	b		
		c	
a	b		
	b	c	
a		c	
a	b	c	

2. Do you agree that the following clinical features as single feature [S] or in combination only [C] are highly suggestive of Glut1D and warrant LP and SLC2A1 analysis?

	[S]	[C]
Paroxysmal eye movements in infancy		
Unexplained paroxysmal events (any age)		
Developmental delay otherwise unexplained		
Effective seizure control by KD in children with intractable epilepsy		
Early-onset intractable childhood epilepsy unresponsive to AEDs		
Early-onset absence epilepsy		
Myotonic-atonic epilepsy (Doose Syndrome)		
Any complex movement disorder with spasticity, dystonia, ataxia as predominant features		
Alternating hemiplegia		
Stroke-like episodes		
Other:		

3. diagnostic workup for Glut1D (not for introduction of KD!) should include:

		I agree
Blood	Basic lab (renal/liver/thyroid function)	
	Full blood count	
	lactate	
	Amino acids	
	Fasting lipids (TCHOL, TG, HDLC, LDLC)	
Urine	Amino acids, organic acids	
Others	EEG	
	Fasting EEG	
	Cranial MRI	
	Cranial PET	
Genetics	SLC2A1 sequencing	

4. KDTs in Glut1D should be started as early as possible. Yes/No

5. The higher the ketosis, the more beneficial a KDT in Glut1D. Yes/No

6. In Glut1D patients beyond infancy with generalized seizures (excluding absence epilepsy) unresponsive to KD please rank the following AEDs you apply in your center:

RANK (1-5)

- valproate (in girls not beyond puberty) ....
- lamotrigine ....
- ethosuximide ....
- levetiracetam ....
- other (only 1 AED): ....

7. In Glut1D patients with movement disorders/paroxysmal events unresponsive to KDT do you recommend an individual trial of
   - Alpha-lipoic acid Yes/No
   - Triheptanoin Yes/No
   - acetazolamide Yes/No

8. I agree with the following statements of the 2018 KD consensus guidelines on the use of KD:

- KDTs are the treatment of first choice for Glut1D Yes/No
- Introducing and maintaining KDTs for Glut1D does not differ from general recommendations for management of KD's as described in Epilepsia Open. 2018 May 21;3(2):175-192. Yes/No
- Adverse effects of KDT in Glut1D do not differ from KDT for intractable epilepsy and require similar monitoring. Yes/No
- The MAD is used effectively in adolescents, and adults with Glut1D and provides a good alternative to the classic KDT Yes/No
- The MAD can be considered for a school-aged child with Glut1D if the parents refuse the KD Yes/No
- Adults with Glut1DS also benefit from KDT and should be encouraged to try it Yes/No
- The LGIT should currently not be used in Glut1D Yes/No
- Glut1D patients with epilepsy should be trialed on KDT, not on alternative options (e.g. ketoesters) without KDT? Yes/No
- Glut1D patients with movement disorders should be trialed on KDT, not on alternative options without KDT? Yes/No

9. All patients on KDT with Glut1D should have carnitine levels checked regularly? Yes/No

10. All patients on KDT with Glut1D should be on daily carnitine regardless of levels Yes/No

X. Any other important questions for discussion at meetings? (fill in please)

....

Thanks!!

Joerg Klepper and Eric Kossoff
Contact: joerg.klepper@klinikum-ab-alz.de
